# Family Systems Care ‒ Expert consensus on ethics behind committed practice

**DOI:** 10.1177/09697330251339060

**Published:** 2025-06-07

**Authors:** Corina Sgier, Margrit Hilpertshauser, Mirjam Mezger, Melanie Werren

**Affiliations:** 156378 Institute of Biomedical Ethics and History of Medicine (IBME); Zurich University of Applied Sciences; Zurich University of Applied Sciences

**Keywords:** Family Systems Care, Healthcare Professionals, Calgary models, Illness Beliefs Models, Delphi Method

## Abstract

**Background:**

Ethically challenging situations often arise in interactions between healthcare professionals (HCPs) and families. Although the Calgary models offer guidance for practical work and are beneficial in various challenging situations, explicit awareness and recognition of the ethical concepts and theories underlying Family Systems Care (FSC) is generally lacking.

**Method - Research aim:**

This study examined the basis of FSC in virtue ethics, deontology, and teleology.

**Research design:**

Utilizing a qualitative design, an expert consensus was conducted through two focus group interviews, a Delphi group, and an expert panel.

**Participants and research context:**

The expert consensus consisted of 23 professionals in FSC from various specialities, including nurses (*n* = 18), midwives (*n* = 4), and a general practitioner, who explored ethical considerations in clinical practice.

**Ethical considerations:**

The research project was conducted in accordance with the ethical principles of participants’ informed consent and the Declaration of Helsinki.

**Findings:**

Participants recognized the significance of classical virtues such as faith, fortitude, hope, and caritas in FSC. They emphasized that these virtues not only guide HCPs in their practice but also empower families to rediscover their strengths amid suffering. Additionally, the integration of deontological principles and teleological perspectives highlighted the importance of balancing individual and collective well-being, and fostering compassionate relationships while navigating ethical complexities in therapeutic conversations.

**Conclusion:**

This study highlights the importance of virtue ethics, deontology, and teleology in guiding HCPs’ moral reasoning within FSC. Participants emphasized respect and appreciation as essential values for maintaining trust with families during ethical challenges. By integrating ethical theories into practice, HCPs can navigate complex situations effectively, fostering compassionate and dignified care.

## Introduction

Interactions between family members and healthcare professionals (HCPs) in clinical settings can lead to challenging situations where ethical theories are either intentionally applied or implicitly relevant. In daily clinical practice, ethical conflicts may cause HCPs to distance themselves from families.^1,2^ Such conflicts often precede moral distress^3^ and can lead to physical, psychological, social, and spiritual suffering among nurses, ultimately resulting in burnout.^4^ In 1998, Tuckett described the challenge for nurses in particular to find a balance between responding ‘with a sense of compassion’ and ‘adhering to a rule at all costs’ (*p.225*). Based on virtue theory, utilitarianism, and deontology, he outlined a conceptual ethical framework for nurses in practice.^5^ These ethical approaches are essential for developing ethical awareness in nursing and support HCPs in making informed decisions that respect both patient needs and professional standards.

As general guidelines and thus also for ethical orientation in both standard and advanced family nursing practice, the most widely used set of models is the ‘Calgary models’,^6^ pioneered by Lorraine M. Wright, Maureen Leahey, and Janice M. Bell for Family Systems Nursing. These models are multidimensional with the aim to maintain, promote, or restore the health of the family.^8^ Structural, developmental, and functional aspects of the family situation (CFAM) are used to identify strengths and resources, as well as difficulties and illnesses.^6^ The CFIM offers cognitive, affective, and behavioural interventions for nursing conversations with families.^6^ Through commendations and circular questions, CFIM enables nurses to facilitate a context in which the family and its members can find their own solutions to problems.^8^ The IBM addresses the illness beliefs of patients, family members, and HCP and their role in promoting family health.^7^

Although Wright et al.^6^ and Wright and Levac^9^ have mentioned ethical and theoretical concepts, there appears to be a lack of active mention and explicit awareness of the ethical concepts and theories underlying Family Systems Care (FSC). While several studies address specific ethical issues in the application of FSC,^8,10–13^ its grounding in ethics remains only vaguely described. Burchard,^8^ for example, argues that engaging with ethical considerations can enhance the conceptual understanding of FSC and increase the awareness of relationship-oriented work with families. This is essential for nurses who engage with families on a daily basis and highlights the need for systematic reflection on ethics among HCPs working with FSC. While Burchard^8^ discusses the connection between teleology and the FSC, however – two principal areas of ethical thought crucial to nursing practice – virtue ethics and deontology were not considered.

### Research aim

Through exploration in expert consensus, this study seeks to identify and deepen the understanding of the ethical dimensions that underpin the application of FSC in clinical settings, focussing on virtue ethics, deontology, and teleology as central ethical theories already recognized as relevant to good nursing practice. The FAMETHI project (FAMily System Care and ETHIcs) addresses the question of ethically challenging situations in working with families, the ethical philosophies considered normative by HCPs, and how this knowledge can enhance family-focussed, systemic daily work and multilateral family counselling. This article focuses on ethical theories – virtue ethics, deontology, and teleology – based on the practical experiences of HCPs involved in the project, while additional topics are covered in a supplementary paper.

## Methods

### Research design

The FAMETHI research project utilized expert consensus from professionals with experience in Family System Care (FSC) to explore the intersection of FSC and ethics. A qualitative design was employed, incorporating focus group interviews, a Delphi process, and expert panel meetings to gather diverse perspectives.

### Participants and research context

The participants (*N* = 23) were nurses, midwives, and a general practitioner from various specialities (see Table 1). An initial 90-min focus group interview was conducted to identify ethical challenges in clinical practice. Participants were then divided into a Delphi group (*n* = 19) and an expert panel (*n* = 4).

**Table 1.** Participants and research context.

Research context		Participants (N = 23) nurses (n = 18), midwives (n = 4), general practitioner (n = 1)	
Study context	German-speaking experts from the fields of FSC and ethics were contacted	Delphi group composition (n = 19)	- Three midwives - One general practitioner - Nursing experts from the fields of: • Neonatology • Pediatrics • FSC (Family Support Care) • Clinical ethics • Acute care • Intensive Care/emergency ward • Psychiatry • Oncology • Long-term care • Palliative care
Expert selection	Convenience sampling (based on availability and accessibility)		
Expert qualifications	- Well-established within the informal FSC network - Engaged in advanced practice within the field of FSC - Completed specialized training and educational programs in FSC or ethics	Expert panel composition (n = 4)	- One midwife - Nursing experts from the fields of: • Pediatrics • FSC (Family Support Care) • Home care services

The Delphi group completed three written anonymous survey rounds (see Table 2), in each of which they received a summary of the expert panel’s ideas and beliefs, followed by specific questions for written feedback. The selection of the Delphi method was justified by its constructivist nature,^14^ which facilitates the systematic aggregation of expert opinions and the establishment of a well-grounded consensus.^15–17^ Given the exploratory character of this study, no predefined criteria for consensus were established.^14^ To reach a consensus, the collective intelligence of the Delphi group was integrated with the expert panel through an iterative process and was subsequently validated internally in a collaborative focus group interview.^14^

**Table 2.** Content of the survey rounds.

Survey round	Ethical perspective	Focus
First	Virtue Ethics	Highlights the importance of beliefs and the sense of correctness in making ethical decisions when working with families, emphasizing the character traits of the individuals involved
Second	Deontological perspective	Focuses on the responsibility of professionals to initiate change. It acknowledges the coexistence of different perceptions of reality, which includes the obligation to fulfil moral principles while respecting the dignity and perspectives of all parties involved
Third	Teleological approach	Emphasizes the consequences and practical impacts of ethical decisions in everyday life, within the structural challenges of the healthcare system

The expert panel (see Table 1) held six 3-hour meetings, starting with the introduction to a specific area of ethics followed by presentations of Delphi group responses. The participants of the expert panel then considered their own experiences from practical work with families in terms of this area of ethics. The introductions to ethical theory were drawn from Dietmar Hübner’s Introduction to Philosophical Ethics,^18^ which provides a clear framework for key ethical concepts and debates. Additional sources included Düwell’s Handbook of Ethics,^19^ Riedel’s Ethics in Healthcare,^20^ Conradi and Vosman’s Practice of Attentiveness,^21^ and various articles from the Stanford Encyclopedia of Philosophy.^22^ Additionally, to establish a connection with FSC, each session included the discussion of appropriate material from the Illness Beliefs Model (IBM),^7^ which was then linked to the ethical concepts. In a concluding focus group interview with all participants, which also lasted 90 minutes, the results were presented for a member check, in which the collected data were critically reviewed, discussed, and further refined. The aim of this study was not to assess the theories for coherence and consistency but rather to explore their relevance to FSC and to gain inspiration from them. Participants concentrated on the elements that were meaningful to them and relevant to their daily work.^23–25^ Data analysis was conducted based on the QUAGOL method^26^; however, the team approach was omitted due to resource constraints.

### Ethical considerations

The research project was conducted in accordance with the ethical principles of participants’ informed consent and the Declaration of Helsinki. According to the Cantonal Ethics Committee (KEK) of the Canton of Zurich, this study does not fall under Swiss legislation regarding human research, as confirmed by the Cantonal Ethics Committee on the December 14, 2021 (waiver no. Req-2021-01424). No participants were at risk of harm by participating and all signed an informed consent form prior to enrolment in the study. The data were collected in accordance with the ethical principles of informed consent, confidentiality, and the participants’ right to withdraw at any time without giving reasons. There were no conflicts of interest.

## Findings

Family Systems Care (FSC) engages with metaethics, particularly through cognitivism and constructivism, to form its ethical foundation for moral guidance.^6^ In FSC, cognitivism holds that moral values are not subjective or relative but emerge from biological and biographical factors, suggesting an objective moral truth, though not readily apparent.^1^^6,9^ Constructivism, aligned with pragmatism and communication theories, argues that moral norms are created, not discovered, based on biological structures. It emphasizes that our perceptions of reality are socially constructed, influenced by individual and social processes.^18^ This view opposes a purely scientific approach, promoting a pluralistic understanding of ethics that considers relational dynamics and contextual factors, rather than focussing solely on objective truth. Social constructivism, rooted in 1980s psychology, further underscores that knowledge. Ethical norms are shaped by social and cultural contexts.^6^ In FSC, this relational focus shifts attention from isolated individuals to the interactions and relationships within families, suggesting that ethical decision-making is a product of ongoing, dynamic exchange. This constructivist perspective helps to inform healthcare professionals’ (HCPs) engagement with families, allowing for more flexible and responsive care that integrates both scientific knowledge and individual perspectives.

In ethically challenging situations, HCPs in FSC identified three main concerns: recognizing ethical dilemmas, applying ethical reasoning, and implementing decisions in practice. The most challenging ethical aspects in day-to-day practice is having to implement ethical decisions without being involved in the decision-making process, managing difficult communication, and navigating contextual limitations that contradict personal beliefs. Participants reported using three approaches to address these dilemmas: a pragmatic-intuitive response, an emotional approach, and a cognitive performance of ethical discourse. While emotional and intuitive responses often stem from lived experience, ethical theories helped HCPs articulate their thoughts and better communicate their concerns. Furthermore, participants emphasized that ethical awareness should be present in all interactions with families, not just in ethically challenging moments. Ethical engagement requires empathy, presence, and an open attitude, while a balance between emotional and cognitive approaches is crucial. In resource-constrained environments, HCPs often face conflicts between professional beliefs and practical limitations, as seen in cases like late-term abortions and patients' requests for suicide. Although ethical theory helped express intuitions more clearly, participants stressed the importance of an empathic and respectful attitude in everyday family interactions. This combination of intuitive, emotional, and cognitive strategies helps navigate the complexity of real-world ethical dilemmas.

During data collection, it became clear which ethical theories were most relevant to HCPs in their clinical work with families. This section outlines the areas of virtue ethics, deontology, and teleology in relation to the practical experiences of participants, as these three proved central to the discussion of morally informed decision-making. Further topics that emerged from the participants’ experience within FSC in relation to ethical thought are dealt with in a companion paper.

### Virtue ethics

**Figure 1.**
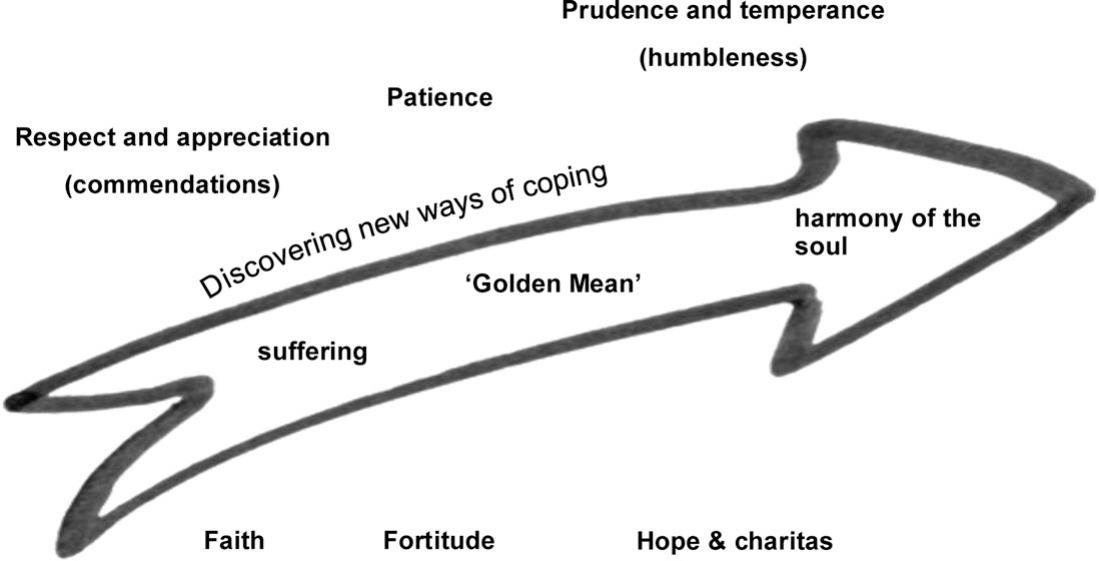
Practical relevant aspects of virtue ethics (Source: Created by the author).

Virtue ethics originated in antiquity and is the oldest moral philosophical approach in Western philosophy. A virtue is a fixed disposition of character that enables human beings to perform the right action under appropriate circumstances.^18^ Perspectives on virtue introduced to the expert panel included those of Plato, Aristotle, Thomas Aquinas, and more modern philosophers such as Alasdair MacIntyre and Martha Nussbaum. The expert panel explored several virtues based on the theoretical introduction and deduced which they consider significant in therapeutic conversations with families. As illustrated in Figure 1, the participants singled out classical virtues such as faith (fides), fortitude (fortitudo), hope (spes), and ‘caritas’ in discussion. The values of respect and appreciation – often expressed through commendations in the FSC – were also emphasized, with participants perceiving them almost as virtues. The virtues patience, as well as prudence (prudentia) and temperance (temperancia) referred to as humbleness (humilitas) in FSC. All these virtues were perceived by participants and seen to have the potential to assist individuals and families in transcending suffering, guiding them from the ‘Golden Mean’ towards Plato’s ideal of soul harmony,^18^ as they discover new paths for coping and healing.

Aristotle describes each virtue as existing on a continuous scale between lack and excess. The search for a balance between two extremes was evident through all the participants’ discussions, just as Aristotle stipulates a ‘Golden Mean’ as desirable in the exercise of every virtue. The ‘Golden Mean’ emphasizes moderation and balance in ethical behaviour. For example, ‘honesty’ (alētheia) is a virtue. The exaggeration of ‘being honest’, positively intended, can be experienced in its effect as a negative ‘hurtful’ behaviour. Doing the right thing, according to Aristotle, therefore means finding the middle way between exaggeration and deficiency. Aristotle says that the virtue of ‘wisdom’ (*sophia*) allows people to find this ‘Golden Mean’. People learn to do this through experience, reflection, and analysis. Through habituation, repeated practice, a transition takes place from the mind-virtue ‘wisdom’ to the more lived character virtues. Habituation can also go wrong and result in an undesirable tendency towards one extreme.^18^ The expert consultation can itself be taken as an example of the process of experience, reflection, and analysis, as we sought to learn from the knowledge and experience of the participants.

In the expert panel, participants entertained a long discussion on how best to apply virtue ethics in FSC. They concluded that this pertains on two levels: on the one hand as the virtues of the HCPs in their practice, on the other hand as the potential strengths of the family. Furthermore, the participants concluded that, corresponding with Plato’s descriptions of ‘harmony of the soul’ as the goal of virtuous life,^18^ the goal of FSC is always to strengthen the family and soften suffering, and one way to achieve this goal is by focussing on the virtues of the family.

In practice, the participants explore virtues with families (see Table 3) but usually call them ‘families strengths’. Interestingly, the word ‘virtue’ derives from the Latin term ‘virtus’, which in classical Latin means ‘virtue’, ‘valour’, or ‘efficiency’. It in turn derives from ‘vir’, which means ‘man’, and originally referred to the ideal qualities of a Roman citizen, such as courage, strength, and moral excellence. Thus, HCPs assist families in rediscovering their virtues, that is, strengths within themselves.^27^

**Table 3.** Core virtues for Family System Care.

Virtue/concept	Philosophical origin	Application in FSC (Family System Care)
Faith (fides)	Thomas Aquinas	The participants also discussed Thomas Aquinas' idea that the virtues of faith (fides), hope (spes), and caritas (love/charity) are ‘God-given’. Aquinas argues that these virtues are innate to everyone and cannot be learned or developed.^18^ For the participants, the word ‘faith’ proved to be multi-layered in the linguistic usage of the participants. To avoid confusion with the idea of ‘faith in God’, they preferred the more neutral word ‘trust’. While HCPs could accept that a virtue is God-given, they tended to insist that it should be equally available to all humans or, even better, 'refillable’, regardless of whether they personally believe in a deity or not. The participants of the expert panel found the interpretation most attractive that every human being has trust, hope, and love within himself and can rediscover and develop it
Hope (spes)	Thomas Aquinas	Hope stood out as especially important to the participants. They emphasized that, when working with informed individuals, they do not have the right to deny or destroy the hope of an individual or family, even if it is marginally realistic
Caritas (love/charity)	Thomas Aquinas	The virtue ‘caritas’ shows itself in the experts' work with families as devoted interest or benevolent curiosity. Together with the virtues of trust and hope, participants consider this expression of caritas as the foundation idea behind the FSC approach. Applying these virtues in FSC, they succeed in developing a benevolent relationship with the families and, still more, in addressing many problems preventively in this way
Fortitude (fortitudo)	Aristotle, Aquinas	The participants agreed with Thomas Aquinas’ tenet that the basic virtues of ‘caritas’, faith, and hope are best perfected through the virtue of fortitude (fortitudo). Brave and courageous people hold fast to what they have recognized with their reason, even against personal inclinations and desires.^18^ HCPs need to be brave every day. They face stressful situations that challenge them cognitively, physically, and emotionally, still more than would be the case in ‘normal everyday life’. At the same time, HCPs are present every day and accompany families in pleasant and difficult situations. It takes fortitude to endure seeing families who are suffering and to go through challenges and uncertainties side by side with families
Patience	General (not Aristotelian)	Patience is another virtue the participants considered important. Patience and endurance are not Aristotelian virtues in themselves, but something the participants regarded as necessary in performing FSC
Respect & Appreciation	General (commonly used in FSC)	Respect and appreciation are also not Aristotelian virtues but figured as important in the collaboration of the participants with the family. Both are central values in basic training in FSC but are better known as the practice of commendations. Appreciating and recognizing family and family members' suffering as well as their strengths and hope may be the most powerful interventions used in interactions with families
Temperance (sōphrosynē)	Aristotle, Plato	Two more classic virtues that received considerable attention were wisdom (sophia) and temperance (sōphrosynē), both in connection with the term humbleness used in FSC.^7^ Humbleness is a natural companion of wisdom, as it prevents a person from falling into arrogance or pride. A wise person knows that true knowledge can never be fully attained and is therefore prudent in her judgement and behaviour. Temperance makes it possible to control one's own impulses and inclinations through rational reflection. Humbleness promotes this control by helping us to see ourselves realistically and accept our weaknesses. Both wisdom and temperance aim to recognize and do what is ‘good’. The connection between wisdom, temperance, and humbleness thus lies in recognizing one's own limits and constantly reflecting on one's own actions and thoughts.

HCPs practicing FSC do not see themselves as omniscient experts on families. The participants found that humbleness protects them from unrealizable expectations of themselves. The stance of humbleness gives a framework for the responsibilities participants take in conversations with families. To them, humbelness to them means that they can provide a context for change, but do not hold power over change or the direction of change. This does not absolve them from the responsibility to perform at their best.

Interestingly, the ‘Tree of Virtues’ from the *Speculum virginum* of the 13th century already reflects the ‘Golden Mean’ and the fluid transition between virtues.^28^ This depiction includes the same main virtues that our participants intuitively selected, except for justice (iustitia). Although justice plays a central role in various philosophical traditions – as a cardinal virtue in Plato and Thomas Aquinas, and a character virtue in Aristotle^18^ – it was not addressed by the participants. This may be because they focused on virtues more directly relevant to interpersonal relationships and family support, or perhaps justice was perceived as too abstract, impersonal, or institutional for direct application in therapeutic conversations. It is also possible that the participants do not perceive justice as a virtue, but primarily as a normative value or an ideal worth striving for. Furthermore, in the depiction of the ‘Tree of Virtues’, humilitas (humility in this context also humbleness) is presented as the root of all virtues, a necessary foundation for all others,^29^ aligning with the participants’ insights.

The results show that virtues as character traits are familiar to the participants. They use many different virtues to describe aspects of their FSC practice. They describe how virtues support them in finding the ‘Golden Mean’, in finding arguments in ethical discourse or decisions, and in choosing appropriate and helpful behaviours. Reflecting on virtues can help HCPs in their interaction with family members. Knowing the various forms in which virtues are manifest, participants can discover and elaborate on strengths and resources within a family. Healthcare professionals share the experience that in times of stress and suffering, families often lose access to their own strengths and thus ‘forget’ them. Reconnecting families to their strengths (or virtues) boosts their ability to act and make shared decisions.

### Aspects of deontological theory

Deontology or the ethics of duty and commandments inspired participants to explore the intersection of FSC and Immanuel Kant’s categorical imperative. They found a clear link between the ethical obligations and core beliefs in FSC. What HCPs or families perceived as a duty was often closely aligned with their core beliefs. In the discussion, the concern focused on conflicting obligations that appear to apply at the same time. Moreover, participants identified a connection between the terms ‘duty’, ‘obligation’, and ‘belief’, observing numerous similarities between these concepts. However, the understanding of what constitutes beliefs does not fully align with the concept of duties, as will be further explained below.

Kant’s first formula of the categorical imperative corresponds to the principle of universalizability: ‘Act only according to that maxim whereby you can at the same time will that it should become a universal law’.^30^ In other words, prior to acting, one must consider whether the action would be acceptable if all others acted the same way. In the context of Kant’s argument that moral actions must be based on universal principles, the statement ‘Lying is wrong’. was taken up by the participants. While they agreed with the statement, they also felt the need to qualify it, to add a but-clause as a way of mitigating the sweeping claims. When confronted with the example of hiding refugees from state persecution, for example, the duty to ‘protect life’ was generally considered more important than ‘not to lie’. For Kant, lying remains morally reprehensible even where it might produce an apparently positive outcome, because it violates the universal duty to be truthful. Moral duties are not relative or situation-dependent in his view, but absolute and universally valid. Thus, participants could not unreservedly agree with the ethics of duty, in which moral actions are judged as right or wrong, based on whether they are consistent with moral duties, rather than on their consequences. In contrast to Kant, the participants perceived duties and their hierarchical prioritization as not solely dictated by reason, but also as influenced by contextual factors and frequently intertwined with empirical requirements encountered in everyday life. Furthermore, the situational independence advocated by Kant is viewed in a fundamentally different way within FSC, as evident in the strong focus on contextualization.

As understood within FSC, ‘context’ encompasses the entirety of the situation as well as the background factors that are pertinent to a specific event or individual.^6^ It also refers to context in conversations, creating an open space, for example, by consciously focussing on building authentic, compassionate relationships and getting to know the family in an inquisitive, respectful and non-judgemental way, which makes changes possible.^7^ By creating this context, HCP can facilitate a shift in the personal perspective of the individual holding a specific belief. In one example from a therapeutic conversation, a mother expressed the belief, ‘I must not show my grief to my 13-year-old daughter so as not to burden her’. The mother wanted to protect her daughter, but thereby undermined the connection between them. After a few conversations, the mother’s understanding of the situation changed, and so did her behaviour. The goal of protecting her daughter remained prominent, as not to ‘let all the emotions rain down on her’. However, her priority became ‘being a good mother’, that is, her motivation shifted from a negatively charged to one that is positive and affirming. By exploring her core belief, the mother was able to give a different meaning to a challenging situation and thus to free herself from the worry of ‘overburdening her daughter’. In this way, participants may transition from a deontological perspective that emphasizes contextual independence to an attitude determined by the principles of virtue ethics. In this instance, the focus is on demonstrating how suffering can be alleviated through the modification of beliefs, thereby creating a context in which change is feasible.

While discussing Kant’s principle of universalizability, the participants discovered a similarity to relative autonomy. According to this principle, one’s own autonomy only extends so far as it does not restrict that of others. Relative autonomy,^31^ that is, the ability of an individual to make decisions and carry out actions that are independent but are influenced or restricted by social, cultural, or situational conditions, emphasizes the interplay between personal freedom and contextual factors.

Furthermore, Kant’s assertion that there is a difference between the way individuals present themselves to the external world and their internal feelings^18^ was also reflected in the participants’ statements that they use embodied reactions to recognize when they are approaching core beliefs. By paying close attention to embodied reactions such as facial expressions, gestures, and posture, they ensure that their interpretations are not solely influenced by verbal responses. In their opinion, personal behaviour must be chosen in such a way that it is also attributable to others, but it must not ‘be imposed on’ others. To avoid imposing their own maxims on others, participants expressed a conscious willingness to critically scrutinize their own convictions and sense of duty.

Kant’s second formula of the categorical imperative is: ‘Act in such a way that you treat humanity, whether in your own person or in the person of any other, always at the same time as an end, never merely as a means’.^30^ All participants agree and are familiar with the prohibition of instrumentalization and recognize that human beings must never be merely a means to an end. This aspect was self-evident to the participants, and thus rarely addressed, reflecting the belief in FSC that demonstrating respect and appreciation for others is one of the most crucial interventions.

### Aspects of teleological theory

In teleology, the focus is on the aim to be achieved. Utilitarianism is the best-known form of teleology.^18^ Participants were familiar with utilitarianism and its goal to achieve the greatest possible benefit for the greatest possible number of people.^18^ Tension remains in the participants’ discussions of the proper balance between benefit to the individual and benefit to the entire family, such that the well-being of the individual is not sacrificed to the happiness of all others. Nonetheless, the basic idea of selflessness and prioritizing the community appealed to the participants, which contrasts with the general emphasis on individualism in the western world.

The participants were introduced to the fundamentals of teleological ethics, followed by examples drawn from Hübner.^18^ Surprisingly, the participants focused on statements from philosophers typically associated with teleological views; however, the examples they chose and their contributions were more closely aligned with virtue ethics.

An idea taken from an author typically associated with teleology that caught participants’ attention was Adam Smith’s ‘impartial spectator’.^18^ According to Smith, the moral sense of most people corresponds to the attitude of an ‘attentive spectator’. This observer is characterized by the fact that he has compassion (sympathy) for others and can put himself in others’ shoes. According to Smith, however, compassion is not enough to make morally correct judgements. These also require the perspective of an ‘impartial spectator’, a kind of moral counsellor who helps people to judge their own actions and moral decisions and motivates them to make fairer, more just decisions by forcing them to look at actions from an impartial point of view. Smith assumes that we can all feel a certain sympathy for other people in the sense of compassion for the emotional reactions of others, but also stipulates that empathy alone is not enough to take a moral attitude. While empathy is valued as such, to take a moral attitude, it must be subordinated to a good dose of self-control.^18^ A balance between benevolence and distance maximizes overall benefit. By initially focusing on benevolence, one acknowledges that the primary concern is the well-being of individuals, while maintaining a distance from those affected enables one to view them as a cohesive unit from the perspective of the ideal observer.

The participants derived two useful lessons from the impartial spectator. As a first lesson, the participants replaced the impartial, distanced attitude described by Smith with a ‘multi-partial’ perspective, as delineated by Wright and Bell.^7^ This approach fosters a benevolently curious disposition towards all individuals involved simultaneously. Rather than adopting a neutral or impartial stance, it assumes a ‘*multi-partial*’ attitude, offering equal consideration to all parties. Another discussion centres on the opposition between closeness and distance. Participants describe their understanding of human beings as fundamentally social and relational beings, whereas among HCPs, there is a tradition of distancing oneself and of exercising self-control over all emotions or affections when working with patients. For the participants, this distanced, self-controlled attitude does not do justice to the complex realities of human encounters and runs the risk of indifference towards the other. They prefer to be professionally engaged with fellow human beings. Especially in the early years of a HCPs career, it is challenging to achieve a balance between dedicated and distanced behaviours and attitudes, reaching a form of professional involvement that is neither cold or disinterested nor overly burdened by feelings of responsibility for the patients. Here again, one observes the participants searching for the ideal balance, or Golden Mean, needed to be able to accompany family members with genuine professional commitment.

**Figure 2.**
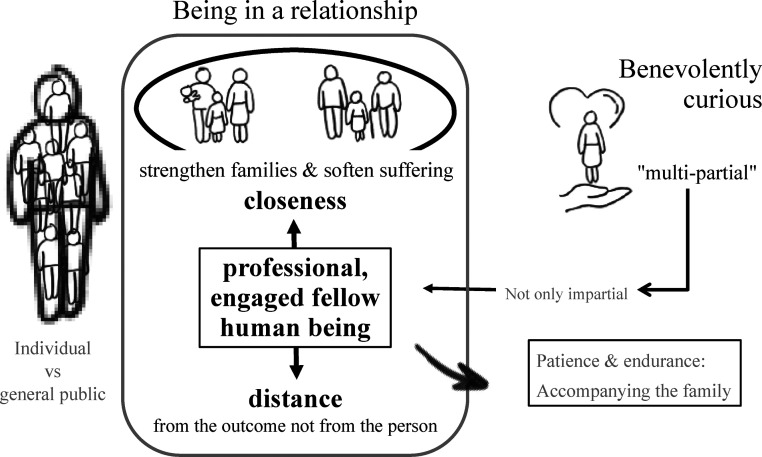
One individual between others (Source: Created by the author).

Strategies identified by the participants to balance distance and closeness with the family members include cooperation in expert teams, clinical reasoning, talking about their hypotheses and possible obstacles, and a clear understanding of their role and relationship in the family conversations. When participants distance themselves, they distance themselves from the outcome but never from the individual. The participants do this in continuous awareness that they do not have the power to effect change for the family on their own, but that they can create the context and the space for this to occur. This relieves the participants and enables them to patiently accompany families in difficult situations over a longer period. Figure 2 schematically illustrates the tensions between individual and collective well-being, the balance of closeness and distance, and the shift from impartiality to considering all involved parties, with the overarching aim of ensuring that families are supported by professionally engaged practitioners over the long term.

## Conclusion

In the course of this study, it became evident that the practice within Family Systems Care (FSC) extends beyond the formal application of the Calgary models. Expert consensus emphasized the fundamental principles of attitude and behaviour as a guiding framework for communication. Nevertheless, the specific components of the Calgary Family Assessment Model (CFAM) and the Calgary Family Intervention Model (CFIM) remain integral to enriching systemic and multilateral family counselling. While no extensive empirical research explicitly addresses ethical reflection within CFAM or CFIM, existing studies^32,33^ indicate positive effects on nursing practice and implicitly considers ethical aspects.

For healthcare professionals (HCPs) engaged in FSC, ethical practice is aptly encapsulated in the words of Wright and Bell^7,34^: ‘To be a “particular kind of person” and a way of being in clinical practice are represented by a person who looks for strengths amid suffering, hope amid despair, and meaning amid confusion’ (page 303). This characterization illustrates that the attitudes and ethics underpinning FSC cannot be confined to a singular theoretical framework. Rather, they embody a relational dynamic within the HCP-family interaction, wherein the cultivation of ‘goodness’ facilitates the pursuit of equilibrium or ‘the middle’. HCPs endeavour to foster an environment that recognizes and nurtures strengths, promoting resilience and connection while facing challenges.

By offering an overview of ethical concepts and reflecting on expert consensus, we identified the contribution of virtue ethics, deontology, and teleology within FSC and deepened appreciation of their significance and reveal their utility.

Virtue ethics inform participants in creating a context of respect and appreciation, enabling them to stay present and in touch even when ethical challenges arise. Participants describe a trusting relationship as the most ethical way of working with families. This way of working likewise finds expression in the IBM.^7^ Wright offers a formula that expresses the participants’ experience with virtues: ‘Kindness, compassion, and love are healing’,^35^ corresponding to caritas and humility. While engaging with the examples provided from authors in the realms of deontology and teleology, the participants tended to discuss aspects more closely aligned with virtue ethics. It is possible that virtue ethics resonated more with them, as they may perceive it as more immediately applicable and adaptable to practical situations.

Closely related to virtues is the deontological approach, emphasizing recognizing human dignity and treating patients with moral care as a duty. While participants did not fully align with Kant’s ethics of duty, they found similarities between duties and beliefs. FSC imposes an obligation to value others, recognize perspectives, and respond appropriately. As Tuckett noted,^5^ deontological theory does not clarify how an individual should act when faced with conflicting but equally binding obligations. He suggests that caregivers are guided by deontological principles, acting from a sense of duty and adhering to generally accepted rules.^5^ The participants expanded on the idea, finding that, in the context of the FSC, duty may correspond to principles that encourage engaging with families in a non-judgemental manner, adopting a curious and benevolent attitude, and being aware of other people’s needs and perspectives in relation to relative autonomy.

Teleological perspectives were again characterized by virtue ethics. The balance between empathy and self-control is also thematized in Smith’s theory. Participants achieve this balance through a multi-participatory attitude. This approach fosters collaboration, focuses on families’ concerns, and removes obstacles in the relationship.^36^ The goal of the FSC to empower families and alleviate suffering is achieved, in part, through a multilateral approach that engages with each individual with benevolent curiosity while also considering the well-being of the family as a whole. This aligns ethically with a utilitarian perspective.^18^ Caregivers guided by utilitarian principles strive to maximize positive outcomes for the entire family while promoting individual well-being.^5^

This study highlights FSC’s profound ethical foundation. Expert consensus reinforces the significance of the Calgary models and underscores integrating fundamental principles at all levels of education. A deeper exploration of FSC and ethics enhances understanding, strengthens personal behaviour, and builds confidence. Raising awareness of these connections enriches family practice in multiple ways (see Table 4). Although many considerations are embedded in FSC’s foundations, this study enhances awareness of strengthening personal conduct and confidence. It underscores the importance of balance in all aspects of practice. By integrating these insights, HCPs can better address individual and family needs while navigating ethical challenges with clarity and compassion.

**Table 4.** Practical implications of ethical approaches in Family Systems Care: enhancing respect, dignity, and balance.

Practical implications	Ethical perspective	Key focus
Cultivating a Respectful and Appreciative Environment	Virtue Ethics	Applying virtue ethics can enable counsellors to create a context characterized by respect and appreciation. This approach facilitates to remain present and stay in contact with family members, even in ethically challenging situations. It also emphasizes the importance of context and relationship building
Commitment to Recognizing the Dignity and Perspective of Each Individual	Deontological perspective	The duty to recognize each individual’s dignity and treat people with respect and moral care entails considering account the perspectives of all family members. Adopting a non-judgemental, benevolently curious attitude is therefore essential
Striving for Balance and the Middle Path	Teleological approach	A new aspect seems to be the pursuit of balance and the middle path as a goal. This approach highlights for example the equilibrium between benevolence and distance. Achieving this balance can be facilitated through a ‘multi-partial’ perspective, allowing for the development of cooperative relationships and a focused, open engagement with family concerns while maintaining professional distance. Viewing the family as a dynamic system, where individual parts and the whole are interrelated, is pivotal. Addressing both individual and collective well-being aligns with a utilitarian perspective

Our participants were advanced with a clear ethical perspective. Since most HCPs apply FSC based on foundational training rather than advanced practice, findings may have limited applicability to family nursing. Another limitation is potential priming bias from presented content. Despite efforts to ensure clarity and coherence, presenting discussion material inherently introduces bias.

Overall, findings support the integration of ethical theory into clinical practice, as it can shift perspectives, beliefs, and attitudes, enabling HCPs to apply FSC principles more effectively and navigate challenging situations more successfully. The ‘Golden Mean’ must always be sought – whether between extremes (as with virtues), closeness and distance, different family members, or theory and practice. This interaction between beliefs, FSC and ethical theories occurs across all levels.

## Data Availability

The datasets used and analysed during the current study are available from the corresponding author on reasonable request.*

## Note

1. Moral values are not merely subjective opinions, as suggested by subjectivism, but are grounded in an objective foundation—namely, in the human condition itself, shaped by both biological and biographical factors—and are therefore not simply culturally constructed, as relativism would propose. The constructivist approach within FSC can thus be most closely aligned with cognitivism, as it acknowledges the truth-apt nature of moral judgments, even if their formation is influenced by social and cultural processes.

## References

[1] Varcoe C Pauly B Storch J , et al. Nurses’ perceptions of and responses to morally distressing situations. Nurs Ethics 2012; 19(4): 488–500. 22619236 10.1177/0969733011436025

[2] Wiegand DL Funk M . Consequences of clinical situations that cause critical care nurses to experience moral distress. Nurs Ethics 2012; 19(4): 479–487. 22619234 10.1177/0969733011429342

[3] Falcó-Pegueroles A Lluch-Canut T Guàrdia-Olmos J . Development process and initial validation of the ethical conflict in nursing questionnaire-critical care version. BMC Med Ethics 2013; 14: 22. 23725477 10.1186/1472-6939-14-22 PMC3711987

[4] Rushton CH Batcheller J Schroeder K , et al. Burnout and resilience among nurses practicing in high-intensity settings. Am J Crit Care 2015; 24(5): 412–420. 26330434 10.4037/ajcc2015291

[5] Tuckett AG . An ethic of the fitting: a conceptual framework for nursing practice. Nurs Inq 1998; 5(4): 220–227. 10188483 10.1046/j.1440-1800.1998.00241.x

[6] Wright LM Leahey M Shajani Z , et al. Familienzentrierte Pflege : Lehrbuch für Familien-Assessment und Interventionen. 3., vollständig überarbeitete und erweiterte Auflage ed. Bern: Hogrefe, 2021, p. 368.

[7] Wright LM Bell JM . Illness beliefs: the heart of healing in families and individuals. Middletown: 4th Floor Press, Inc., 2021.

[8] Burchard DJ . Ethos, ethics, and endeavors: new horizons in family nursing. J Fam Nurs 2005; 11(4): 354–370. 16287836 10.1177/1074840705281750

[9] Wright LM Levac AM . The non-existence of non-compliant families: the influence of Humberto Maturana. J Adv Nurs 1992; 17(8): 913–917. 1506541 10.1111/j.1365-2648.1992.tb02018.x

[10] Bagnasco A Cadorin L Barisone M , et al. Ethical dimensions of paediatric nursing: a rapid evidence assessment. Nurs Ethics 2018; 25(1): 111–122. 27005952 10.1177/0969733016631161

[11] El Ali M Licqurish S O'Neill J , et al. Truth-telling to the seriously ill child - Nurses' experiences, attitudes, and beliefs. Nurs Ethics 2024; 31(5): 930–950. 38128903 10.1177/09697330231215952 PMC11370149

[12] Kim H Deatrick JA Ulrich CM . Ethical frameworks for surrogates' end-of-life planning experiences. Nurs Ethics 2017; 24(1): 46–69. 27005954 10.1177/0969733016638145 PMC5033678

[13] Vasconcelos SC Silva AO Moreira MASP , et al. Bioethical analysis to the therapeutic use of Cannabis: integrative review. Nurs Ethics 2019; 26(1): 96–104. 28514883 10.1177/0969733017703699

[14] Jünger S Payne SA Brine J , et al. Guidance on Conducting and REporting DElphi Studies (CREDES) in palliative care: recommendations based on a methodological systematic review. Palliat Med 2017; 31(8): 684–706. 28190381 10.1177/0269216317690685

[15] Häder M . Delphi-Befragungen. Wiesbaden: VS Verlag für Sozialwissenschaften, 2009, p. 244.

[16] Nasa P Jain R Juneja D . Delphi methodology in healthcare research: how to decide its appropriateness. World J Methodol 2021; 11(4): 116–129. 34322364 10.5662/wjm.v11.i4.116 PMC8299905

[17] Shang Z . Use of Delphi in health sciences research: a narrative review. Medicine (Baltim) 2023; 102(7): e32829. 10.1097/MD.0000000000032829 PMC9936053 36800594

[18] Hübner D . Einführung in die philosophische Ethik. Göttingen: Vandenhoeck & Ruprecht, 2021, p. 283.

[19] Düwell M Christoph H Werner MH . Einleitung. Ethik: Begriff – Geschichte – Theorie – Applikation. In: Düwell M Christoph H Werner MH (eds) Handbuch Ethik. 3. aktualisierte Auflage. Stuttgart: J.B. Metzler, Part of Springer Nature - Springer-Verlag GmbH; 2011.

[20] Riedel A Lehmeyer S . Ethik im Gesundheitswesen. Berlin, Heidelberg: Springer Berlin Heidelberg, 2023.

[21] Conradi E . Takecare. Grundlagen einer Ethik der Achtsamkeit. Frankfurt/Main: Campus Verlag, 2001.

[22] The Stanford Encyclopedia of philosophy. Stanford University, n.d. Available from: https://plato.stanford.edu/

[23] Schütze F . Biographieforschung und narratives Interview. Neue Praxis 1983; 13(3): 283–293.

[24] Rosenthal G . Biographieforschung. In: Baur N Blasius J (eds) Handbuch Methoden der empirischen Sozialforschung. Wiesbaden: Springer Fachmedien Wiesbaden, 2014, pp. 509–520.

[25] Küsters I . Narratives interview. In: Baur N Blasius J (eds) Handbuch Methoden der empirischen Sozialforschung. Wiesbaden: Springer Fachmedien Wiesbaden, 2014, pp. 575–580.

[26] Dierckx de Casterlé B Gastmans C Bryon E , et al. QUAGOL: a guide for qualitative data analysis. Int J Nurs Stud 2012; 49(3): 360–371. 21996649 10.1016/j.ijnurstu.2011.09.012

[27] A Latin dictionary. Oxford: Clarendon Press; 1879. Virtus.

[28] Powell M . ‘Paradisum speculatorium in picturam ponere’: developing a picture program as the ‘Mirror of Virgins’, 2020, pp. 123–156.

[29] Castelberg M. (2014). *Wissen und Weisheit: Untersuchungen zur spätmittelalterlichen, Süddeutschen Tafelsammlung' *(Washington, D.C., Library of Congress, Lessing J. Rosenwald Collection, ms. no*. 4)*. Berlin, Boston: De Gruyter. 10.1515/9783110332643

[30] Kant I . Grundlegung zur Metaphysik der Sitten. Translated as groundwork of the metaphysics of morals 1785.

[31] Dworkin G . The nature of autonomy. Nordic Journal of Studies in Educational Policy 2015; 2015(2): 28479.

[32] Firda Laela N Yudhi P Andan F et al. Calgary family intervention model approach to improve quality of life for diabetes mellitus patients. KnE Social Sciences. 2023; 8(4): 1–10.

[33] Mileski M McClay R Heinemann K , et al. Efficacy of the use of the calgary family intervention model in bedside nursing education: a systematic review. J Multidiscip Healthc 2022; 15: 1323–1347. 35734541 10.2147/JMDH.S370053 PMC9208629

[34] Wright LM . Suffering and Spirituality: the path to illness healing. Canada: 4th Floor Press, 2017.

[35] Wright LM . Softening suffering through spiritual care practices: one possibility for healing families. J Fam Nurs 2008; 14(4): 394–411. 19139155 10.1177/1074840708326493

[36] Bell JM . Relationships: the heart of the matter in family nursing. J Fam Nurs 2011; 17(1): 3–10. 21343619 10.1177/1074840711398464

